# Tracking chromosomal positions of oligomers - a case study with Illumina's BovineSNP50 beadchip

**DOI:** 10.1186/1471-2164-11-80

**Published:** 2010-02-01

**Authors:** Armin O Schmitt, Ralf H Bortfeldt, Gudrun A Brockmann

**Affiliations:** 1 Department of Crop and Animal Sciences, Humboldt-Universität zu Berlin, Invalidenstraße 42, 10115 Berlin, Germany

## Abstract

**Background:**

High density genotyping arrays have become established as a valuable research tool in human genetics. Currently, more than 300 genome wide association studies were published for human reporting about 1,000 SNPs that are associated with a phenotype. Also in animal sciences high density genotyping arrays are harnessed to analyse genetic variation. To exploit the full potential of this technology single nucleotide polymorphisms (SNPs) on the chips should be well characterized and their chromosomal position should be precisely known. This, however, is a challenge if the genome sequence is still subject to changes.

**Results:**

We have developed a mapping strategy and a suite of software scripts to update the chromosomal positions of oligomer sequences used for SNP genotyping on high density arrays. We describe the mapping procedure in detail so that scientists with moderate bioinformatics skills can reproduce it. We furthermore present a case study in which we re-mapped 54,001 oligomer sequences from Ilumina's BovineSNP50 beadchip to the bovine genome sequence. We found in 992 cases substantial discrepancies between the manufacturer's annotations and our results. The software scripts in the Perl and R programming languages are provided as supplements.

**Conclusions:**

The positions of oligomer sequences in the genome are volatile even within one build of the genome. To facilitate the analysis of data from a GWAS or from an expression study, especially with species whose genome assembly is still unstable, it is recommended to update the oligomer positions before data analysis.

## Background

High-density genotyping arrays have become established as a valuable research tool in human genetics. Currently, more than 300 genome-wide association studies of humans were published, reporting about 1,000 SNPs that are associated with a phenotype [1]. Also in animal sciences, high-density genotyping arrays are harnessed to analyze genetic variation [2,3]. To exploit the full potential of this technology, SNPs on the chips should be well characterized and especially their chromosomal position should be precisely known. However, this is a challenge, if the SNPs are not so-called reference SNPs and if the genome sequence is still subject to changes. If reference identifiers (rs-IDs) are known, the position of oligomers can be updated comfortably through Biomart [4]. Otherwise, oligomer position tracking is possible via aligning the oligomer sequences to the genome sequence. In this work, we describe such a mapping strategy in detail so that a scientist with moderate bioinformatics knowledge can reproduce it. Furthermore, we present a case study in which we mapped 54,001 oligomer sequences from Illumina's BovineSNP50 beadarray [5,6] to the bovine genome.

## Results

**Figure 1:**
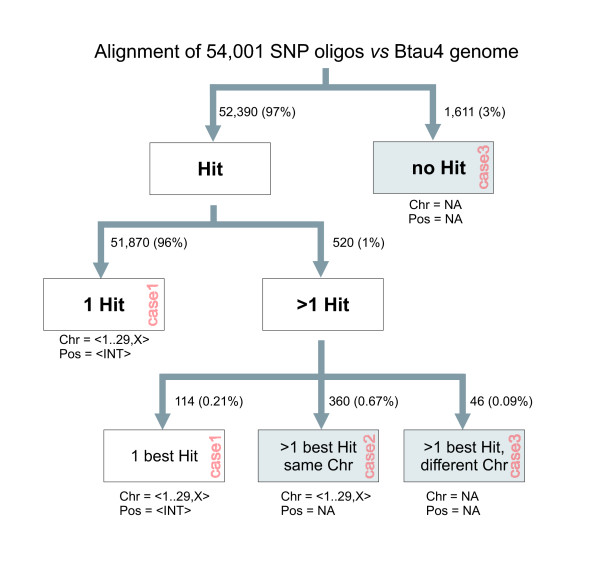
**Evaluation of megablast results for re-mapping SNP oligomers to the Bovine genome**. Grey boxes show filter steps leading to unassigned chromosomes and positions. INT, unique position determined; NA, chromosome or position not assigned, respectively.

51,870 of 54,001 (96%) oligomer sequences had a single megablast hit and could therefore be assigned to both a chromosome and a position unambiguously (Fig. 1). 1,611 oligomer sequences (3%) had no hits, and 520 (1%) had multiple hits. 114 of these 520 oligomer sequences had a clear best hit and could therefore be assigned both to a chromosome and a position. 360 had hits with an equally high bitscore on one and the same chromosome but at different positions. They were assigned to a chromosome but not to a position. 46 had hits with an equally high bitscore but on different chromosomes. They were assigned neither to a chromosome nor to a position. Altogether, we mapped 51,984 (51,870 + 114; 96.3%) oligomer sequences to a unique chromosomal position, 360 (0.7%) to a chromosome but not to a position, and 1,657 (1,611 + 46; 3.1%) neither to a chromosome nor to a position (Fig. 1; Additional file 1: Annotation file for 54,001 oligomer sequences of the Bovine50SNP beadchip). For comparison, the corresponding figures according to Illumina were 52,255 (96.8%), 74 (0.1%), and 1,672 (3.1%) for oligomers with a unique position, for oligomers with a chromosome but without position, and for oligomers without chromosome and position, respectively (Table 1). We wrote an R-script [7] to perform an extensive comparison between Illumina's and our mapping annotation (Additional file 2: Diagnostics.r). In 107 cases, Illumina assigned oligomers to a chromosome, while our mapping procedure did not. In 122 cases, the situation was *vice versa *(Table 2). Next, we examined all 52,222 SNPs assigned to a unique chromosome by both Illumina and our mapping procedure. In five cases (SNP-IDs Hapmap24571-BTA-154685, BTB-01222201, BTB-01798822, INRA-443, and BTB-01386016), the oligomers were assigned a different chromosome by Illumina and our mapping procedure. In 355 cases, SNPs were given a unique locus in Illumina's annotation but not by this mapping procedure; in 74 cases, the situation was *vice versa* (Table 3). We then examined if the positions provided by Illumina and by our mapping procedure differed in case the chromosomal annotation was consistent and, if so, to what extent. 51,788 SNPs had a unique chromosomal position, according to both Illumina and this mapping procedure. In 329 (0.7%) cases, the positions differed by three or more base pairs. In 566 (1.0%) cases, the positions were exactly identical, and in 50,367 (93.3%) cases, the positions differed by one base pair (Table 2). This can probably be attributed to differences in the mapping procedures applied by Illumina and by us. We consider the following cases substantial discrepancies between Illumina's annotation and our results:

**Table 1:** Comparison between Illumina's and this study's annotations.

	Mapped to		
	**unique locus**	**unique chromosome, but no unique position**	**neither unique chromosome nor position**

Illumina	52,255 (96.8%)	74 (0.1%)	1,672 (3.1%)

This study	51,984 (96,3%)	360 (0.7%)	1,657 (3.1%)

Numbers of BovineSNP50 oligomer sequences mapped to the Btau4 genome assembly according to Illumina and according to the re-mapping procedure in this study.

**Table 2:** Chromosomal assignments of SNPs.

This study\Illumina	No chromosome	chromosome
no chromosome	1,550	107

chromosome	122	52,222

Shown are the numbers of SNPs without ("no chromosome") and with ("chromosome") chromosomal assignment according to Illumina and this study.

**Table 3:** Chromosomal positions of SNPs.

This study\Illumina	no position	position
no position	0	355

position	74	51,788

Shown are the numbers of 52,217 SNPs that were mapped to the same chromosome in this study and by Illumina without ("no position") and with ("position") chromosomal position, according to Illumina and this study. In 566 cases, SNP positions were identical, in 50,367 there was a shift of one base pair, in 526 there was a shift of two, and in 329 cases, there was a shift of three of more base pairs.

• Oligomers were mapped to a chromosome by Illumina but not by our mapping procedure (122 cases) or *vice versa *(107 cases)

• Oligomers mapped to one and the same chromosome by Illumina and by our procedure but mapped to a unique locus by Illumina and not by us (355 cases) or *vice versa *(74 cases)

• Oligomers mapped to two different chromosomes by Illumina and by our procedure (five cases)

• Oligomers mapped to one and the same chromosome by Illumina and by our procedure but mapped to *loci *more than two bases apart (329 cases)

Altogether, there were substantial changes for 992 SNPs (1.8%). Finally, we checked if oligomer sequences were represented on the chip more than once (Additional file 3: ComparingOligoSeqs.r). We found 18 cases, where one and the same oligomer sequence was assigned to two different oligomer identifiers (Additional file 4: Eighteen duplicate oligomer sequences). These 18 SNP pairs can be used to assess the genotyping quality. We also compared all oligomer sequences against their reverse-complementary sequences using the R-library seqinr[8] but found no case of duplication.

## Discussion

The underlying genome build was Btau4.0 for both Illumina's annotation and for our mapping procedure, which makes it difficult to find reasons for the discrepancies. One reason could be that minor changes are incorporated into the genome sequence by the genome database curators without altering the build version. Another reason could be that the mapping methods differ. Although the extent of deviations might appear small, incorrect positions of oligomers can lead to artifacts in subsequent analyses like linkage disequilibrium studies or the establishment of candidate gene lists. The given examples illustrate that SNP oligomers that are mapped to different genomic locations may lead to completely functionally different inferences based on gene annotations. It is to be expected that further adjustment is necessary as the bovine genome proceeds to be completed. Currently, 90% of the bovine sequence is assigned to the 29 autosomes and the X chromosome [9].

## Conclusions

Our case study has shown that discrepancies between the initially determined positions and the positions determined at a later time can occur. These discrepancies can make the correct association of an oligomer with a gene difficult. Above all, the classification of an SNP as e.g. non-synonymous coding, synonymous coding or as splice site SNP is critically dependent on its exact chromosomal position.

In a case study with Illumina's BovineSNP50 beadchip we found that almost 2% of the oligomer positions deviated substantially between the annotations given by the manufacturer and determined in our mapping procedure. Given the relatively easy realization of the mapping procedure described here, it is recommended to verify the manufacturer's data and adjust them, if necessary.

We furthermore would like to point out that the verification of results obtained by SNP or expression arrays can be considerably facilitated if the oligomer sequences are made available to the scientific community.

## Methods

We downloaded the bovine genome Btau 4.0 [9] on June 22, 2009 using a Perl-script (Additional file 5: DownloadGenome.pl) to the file GenomeBtauEnsemlb54.fasta. Altogether, the sequences for 29 autosomes, the X-chromosome, one mitochondrial and 11,869 unmapped sequences were downloaded. The bovine Y-chromosome sequence was not available. The total length of the downloaded genome sequence was 2,917,974,530 base pairs, of which 283,544,868 (9.72%) were from unmapped sequences. 108 contig sequences (total length 231,114 base pairs) were not considered, because they were neither assigned to a chromosome nor to an unmapped sequence. Next, we built index files from the genome sequences for the megablast search with the executable formatdb, available from the NCBI C++ toolkit http://www.ncbi.nlm.nih.gov/BLAST/download.shtml. The blast index files were created with the command: formatdb -t "GenomeBtau4" -i GenomeBtau4.fasta -p F -o T -V T -n GenomeBtau4, where the options mean:

• -t: database name

• -i: fasta input file to be formatted into BLAST database format

• -p F: input file containing the chromosome nucleotide sequence

• -V T: check for non-unique string IDs in the database

• -n: base name for BLAST files

We obtained the file containing the oligomer sequences (BovineSNP50_B.csv, last updated on June 4, 2008) from the ftp login, https://www.illumina.com/ftp.ilm. Access to this site is restricted to customers. The file containing the oligomer positions (BovineSNP50_Final_SNPs_54001.zip) was downloaded from http://www.illumina.com/pages.ilmn?ID=256. We will refer to these two files as "oligomer sequence file" and "oligomer position file", respectively. In the oligomer position file, a "0" was interpreted as not assigned to a chromosome or a position. Before obtaining a file containing the oligomer sequences in fasta format, we commented out metadata lines (first seven and last 53 lines) in the oligomer sequence file. The function write.dna from the R-package APE[10] was applied in an R-script (Additional file 6: BuildOligoSeqDB.r) to build a fasta file containing 54,001 oligomer sequences of length 50 base pairs (BovineSNP50OligoSeqs.fasta). In 4,275 cases, oligomer identifiers had to be made consistent. (Oligomer names starting with NFGL-NGS in the oligomer sequence file started with ARS-NFGL-NGS in the oligomer position file). The fasta file was subsequently searched against the bovine genome using the program megablast [11] as follows: megablast -i BovineSNP50OligoSeqs.fasta -d GenomeBtau4 -p 95 -v 1 -b 1 -m 9 -D 3 -F F -o OligosVsBovineGenome.megablast, where the options mean:

• -p 95: identity percentage cut-off 95%

• -v: number of database sequences to show one-line descriptions for

• -b 0: prevent the printing of full alignments

• -F F: do not filter query sequence for low complexity regions

• -m 9: produce tabular output with comment lines

• -D 3: produce tab-delimited output in one line format

• -o OligosVsBovineGenome.megablast: name the output file OligosVsBovineGenome.megablast

**Figure 2:**
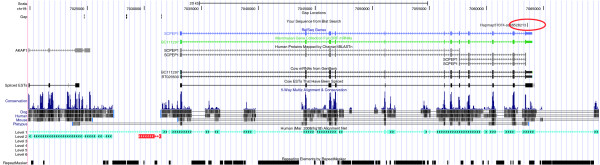
**Example for a unique location**. Example for case 1 in Fig. 1, where an SNP oligomer (Hapmap57074-ss46526213) has a unique locus in the 3'UTR of the *bos taurus *serine carboxypeptidase 1 gene (*SCEP1*) on chromosome 19 (red circle).

**Figure 3:**
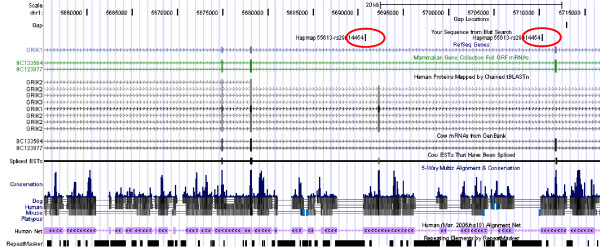
**Example for an oligomer with two *loci *on one chromosome**. Example for case 2 in Fig. 1, where an SNP oligomer (Hapmap55613-rs29014454) maps twice (red circles) between exon 6 and 7 within the *bos taurus *glutamate receptor gene (*GRIK1*) on chromosome 1.

**Figure 4:**
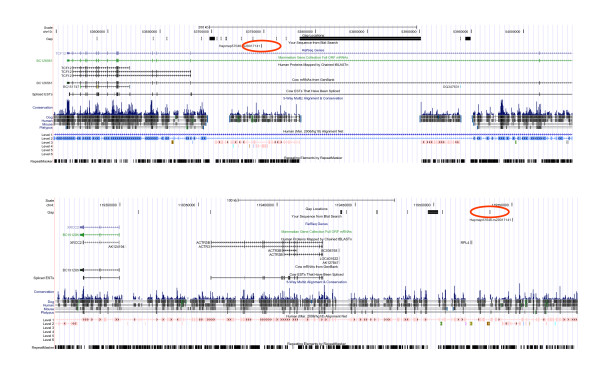
**Example for an oligomer that is mapped on two different chromosomes**. Example of case 3 in Fig. 1, where an SNP oligomer (Hapmap57046-rs29017141) is mapped on different chromosomes: A) between exons 14 and 15 within the *bos taurus *transcription factor 12 gene (*TCF12*) on chromosome 10 and B) within 100 kb downstream of a region on chromosome 4 that closely matches the human homologue of the *ACTR3 *gene.

The output file contained 102,434 hits, *i. e. *about twice the number of oligomer sequences. The megablast output was processed using an R-script (Additional file 7: ProcessMegablastOutput.r) in the following way: Only hits on one of the autosomes or the X-chromosome with an alignment of length 49 base pairs or longer were accepted (53,039 hits). If an oligomer sequence produced just one hit, the position of the end of that hit in chromosomal co-ordinates (plus 1, if oligomer sequence and chromosomal sequence were in the same orientation, otherwise minus 1) was taken as the chromosomal position of the SNP. If an oligomer sequence had no hit, both chromosome and position were assigned NA (not available). In the case of more than one hit, the following strategy of disambiguation was applied: If there was only one best hit of the SNP oligomer in terms of bitscore, its position and chromosome were recorded (case 1 in Fig. 1; Fig. 2). If the hit was neither on an autosome nor on the X-chromosome, NA was assigned to both chromosome and position. Otherwise, if there were several hits with an equally high bitscore on the same chromosome but at different positions that chromosome was assigned to the oligomer, but NA to its position (case 2 in Fig. 1; Fig. 3). Otherwise, if there were several hits with an equally high bitscore on different chromosomes, NA was assigned to both its position and its chromosome (case 3 in Fig. 2; Fig. 4).

## Authors' contributions

AOS designed the strategy, RHB verified the results and tested the scripts, AOS and GAB wrote the manuscript. All authors read and approved the final manuscript.

## Supplementary Material

Additional file 1 **Annotation file for 54,001 oligomer sequences of the Bovine50SNP beadchip**. The column headers designate: ID, ID of the SNP; CHROM.ILMN and POS.ILMN, chromosome and position according to Illumina; CHROM.MAP and POS.MAP chromosome and position as determined in this study. Click here for file

Additional file 2 **Diagnostics.r**. This script compares the original chromosomal positions with those obtained by the mapping procedure described here using the script ProcessMegablastOutput.r. Click here for file

Additional file 3 **ComparingOligoSeqs.r**. This script compares each oligomer sequence against all other oligomer sequences. The aim is to detect oligomer sequences with different IDs sharing the same sequence. Also the reverse complementary sequences are compared against all other oligomer sequences. Click here for file

Additional file 4 **Eighteen duplicate oligomer sequences**. The column headers SNP1 and SNP2 designate the SNPs with identical sequences but different identifiers. CHROM, chromosome of SNP1 and SNP2; POS.ILMN1 and POS.ILMN2, positions of SNP1 and SNP2, respectively, as determined by Illumina; POS.MAP, position as determined in this study. Click here for file

Additional file 5 **DownloadGenome.pl**. This script downloads the most current version of the chromosome sequences of the species specified below from the NCBI database. Look for keyword "Customize". Click here for file

Additional file 6 **BuildOligoSeqDB.r**. This script reads in a comma separated text file containing oligomer sequences and writes out a file containing oligomer sequences in the fasta format. In its present format it is asssumed that the table containing the oligomer sequences has column headers. The column header for the column containing the oligomer sequences is AlleleA_ProbeSeq. This has to be customized if necessary. The ID of the fasta file sequence entries is concatenated from four pieces of information: Name: name of oligomer given by manufacturer; IlmnStrand: orientation of oligomer strand; SourceStrand: orientation of genome strand; SNP: alleles. Click here for file

Additional file 7 **ProcessMegablastOutput.r**. This script reads a file in tabular form that was generated by a megablast search (option D -3 m -9) of oligomer sequences against the whole genome sequence. In its present form it is suitable for the analysis of bovine oligomer sequences of length 50. Click here for file
